# The dilemma of the generalist: expert views on role boundary changes in the NHS and private sector podiatry

**DOI:** 10.1186/1757-1146-8-S1-A1

**Published:** 2015-04-20

**Authors:** Samantha Stressing, Alan Borthwick

**Affiliations:** 1 University of Southampton, Southampton, UK

## Aims/Objectives

In a climate of workforce transition, enhanced specialisation and role transfer, it is timely to address the impact of workforce flexibility on practitioners in non-specialist roles. This study aimed to use podiatry as a case exemplar (as one of the Allied Health Professions) to explore the possible impact of workforce redesign policies on role boundaries on generalist podiatrists and examine the current position of generalist podiatrists in the workforce.

## Content of presentation

The study explored views and experiences of key actors from The Society of Chiropodists and Podiatrists through a qualitative research methodology, incorporating focus groups and in-depth, semi-structured interviews supported by explanatory sociological theory, drawn from the sociology of the professions, workforce literature, and Government policies, noting their impact upon the podiatry profession. Data collected was analysed via a thematic analysis.

## Relevance/Impact

This research is relevant to professionals involved in the SCP, academic departments across the UK and individuals that have influence over policy changes. The findings of this project may also be of interest to those who organise and deliver undergraduate and postgraduate training. This project has added to the evidence base for generalist podiatrists, for which there was no previous research considering the impact of workforce redesign policies on their role. This study has provided an example of how role boundaries can be affected and the perceived impact on the podiatry profession.

## Outcomes

Three themes emerged: the impact of change, concerns about future provision, and meeting the challenge of considering future service provision. The key message was to encourage podiatrists to embrace change by working together, learn from other professions and develop new leaders or ‘champions’ to lead people towards change.

## Discussion

The role of the generalist podiatrist in the NHS may be under threat due to the profession’s focus on the pursuit of specialist ‘virtuoso’ roles, in an attempt to further professionalise. Generalist NHS podiatry is viewed as less of a professional priority, and difficult to sustain and justify in a climate of fiscal restraint. This in turn suggests, therefore, that generalist podiatry may in future become a role entirely associated with private practice.

